# Lack of cognitive impairment in long-term survivors of colorectal cancer

**DOI:** 10.1007/s00520-022-07008-3

**Published:** 2022-04-14

**Authors:** Janette L. Vardy, Gregory R. Pond, Lucette A. Cysique, Thomas M. Gates, Jim Lagopoulos, Corrinne Renton, Louise M. Waite, Ian F. Tannock, Haryana M. Dhillon

**Affiliations:** 1 Faculty of Medicine and Health, University of Sydney, Sydney, Australia; 2 Concord Cancer Centre, Concord Repatriation General Hospital, Hospital Rd, Concord, Sydney NSW 2137 Australia; 3 Centre for Medical Psychology & Evidence-Based Decision-Making, University of Sydney, Sydney, Australia; 4 McMaster University, Hamilton, ON Canada; 5 School of Psychology, University of New South Wales, Randwick, Australia; 6 St. Vincent’s Hospital Applied Medical Research Centre, Sydney, Australia; 7 Brain Mind Research Institute, University of Sydney, Sydney, Australia; 8 Sunshine Coast Mind & Neuroscience, Thompson Institute, University of Sunshine Coast, Birtinya, Australia; 9 Concord Repatriation General Hospital, Sydney, Australia; 10 Princess Margaret Cancer Centre, University of Toronto, Toronto, Canada

**Keywords:** Cognitive function; Survivorship; Colorectal cancer

## Abstract

**Background:**

Our longitudinal study reported cognitive impairment in 43% of people following diagnosis of localised colorectal cancer (CRC) versus 15% in healthy controls (*p* < 0.001) and 50% versus 13% 1–2 years later (*p* < 0.001). Here we evaluate cognitive function and neuroimaging in a subgroup at long-term follow-up.

**Patients and methods:**

Cancer-free Australian participants in the study, and controls, completed cognitive and functional assessments. Neuroimaging was optional. Blood tests included inflammatory markers, clotting factors, sex hormones and apolipoprotein E genotype. The primary endpoint was demographically and practice effect-corrected cognitive scores comparing CRC survivors with controls over time examined using a linear mixed model, adjusted for baseline performance. Secondary endpoints included cognitive impairment rate using the Global Deficit Score [GDS > 0.5], Functional Deficit Score, blood results and neuroimaging.

**Results:**

The study included 25 CRC survivors (60% men, median age 72) at mean 9 years after baseline (9 received adjuvant chemotherapy) and 25 controls (44% men, median age 68) at mean 6 years after baseline. There were no significant differences in cognitive scores or proportion with cognitive impairment (16 vs. 8%) between survivors and controls and no evidence of accelerated ageing in CRC survivors. Baseline cognitive performance predicted for subsequent cognitive function. There were no differences in functional tests or blood tests between groups. In 18 participants undergoing neuroimaging, 10 CRC survivors had higher myoinositol levels than 8 controls, and lower volume in the right amygdala and caudate and left hippocampal regions.

**Conclusions:**

There was no difference in cognitive capacity and function between CRC survivors and controls 6–12 years after diagnosis. Differences in neuroimaging require confirmation in a larger sample.

**Highlights:**

• No evidence of long term cognitive impairment in colorectal cancer survivors compared to controls 6–12 years after diagnosis

• No evidence of accelerated cognitive ageing in colorectal cancer survivors

• No evidence of long-term functional impairment in colorectal cancer survivors

**Supplementary Information:**

The online version contains supplementary material available at 10.1007/s00520-022-07008-3.

## Introduction

Cancer and/or its treatment are associated with cognitive impairment in up to 50% of survivors one year after diagnosis, despite no evidence of cancer recurrence [1–5]. Less is known about the longer-term trajectory of cancer-related cognitive impairment or how it impacts on daily function. Cross-sectional studies have suggested persistent cognitive impairment [6]. For example, a study comparing 196 women with breast cancer who had received adjuvant chemotherapy a mean of 21 years previously to a population-based cohort (*n* = 1509) found that cancer survivors performed less well on verbal memory, processing speed, executive function and psychomotor speed than controls; cancer survivors reported more cognitive symptoms but less depression [7]. It is unclear whether apparent impairments were due to the cancer, chemotherapy or limitations in study design.

Healthy people experience decline in cognitive function, particularly working memory, attention and processing speed, with increased age [8]. Although cancer prevalence increases with age, and many older patients receive chemotherapy, most studies of cognitive function have been in younger women with breast cancer, and few studies have follow-up beyond 1–2 years. Preliminary data suggest that cancer and/or its treatment might accelerate cognitive ageing [9–12].

Our longitudinal prospective study in colorectal cancer (CRC) included men and women, with a median age of 59 years at baseline, with assessments soon after diagnosis, and 6, 12, and 24 months later. We compared cognitive function in survivors with localised CRC (*n* = 291) who did or did not receive neo/adjuvant chemotherapy, and age-matched healthy controls (*n* = 72). At baseline 43% of CRC patients had cognitive impairment compared to 15% of controls [13]. At 1–2 years post diagnosis, 50% of CRC survivors were cognitively impaired versus 13% of controls. There was no significant difference in the prevalence of cognitive impairment between those who did or did not receive chemotherapy, but those receiving chemotherapy had more cognitive decline from baseline [5].

The primary aim of the present study was to evaluate cognitive function in our cohort of CRC survivors 6–12 years after diagnosis. Our a priori hypotheses were that compared to controls, survivors of CRC would have: (i) more cognitive impairment; and, (ii) accelerated cognitive ageing.

## Methods

Methods of the original study are described elsewhere [5, 13], but, in brief, people with CRC were recruited from hospitals in Toronto, Canada, and Sydney, Australia. Controls were recruited from Sydney only, and were generally family or friends of cancer patients. At baseline all participants were ≤ 75 years of age, had no prior invasive malignancy, had no comorbidities likely to cause cognitive dysfunction and could write and speak English. Baseline assessment for those with localised CRC was performed soon after surgery and prior to any chemotherapy. Subsequent assessments were 6, 12 and 24 months later. Controls were assessed at similar times, but not at 24 months.

Australian participants of the original study with no evidence of CRC recurrence or a new primary invasive cancer were contacted by letter or telephone and invited to undergo long-term assessment. The study had Research Ethics Board approval and all participants provided written informed consent.

### Assessments

Participants underwent the following assessments by a trained Research Officer in one or two sessions.

### Neuropsychological assessment

Mini Mental State Examination (MMSE) to screen for dementia.

Wide Range Achievement Test (WRAT) 3 Reading test [14] to assess premorbid ability.

A neuropsychological test battery evaluated four cognitive domains: working memory and attention; processing speed; verbal learning and memory; visual learning and memory (Supplementary Table 1). Testing time ~ 45 min.

Functional Impairment Assessment (FIA) [15] to assess “real worldˮ performance was encouraged but not mandatory. The following skills were evaluated over ~ 1.5 h:

1. *Shopping task* from the *Direct Assessment of Functional Status instrument* (*DAFS*) [16]. Participants recall items from a shopping list and select items from a mock grocery store.
2. *Basic Finances,* from the *DAFS*. Subjects count money, make change from a $5 note, write a cheque, and balance a chequing account.
3. *Advanced Finances*: Subjects are provided with blank cheques, a bank statement, and a calculator: they are instructed to deposit money to an account, pay bills, and leave $100 in the account.
4. *Medication Management*: a revised version of the Medication Management Test^15^ [17], evaluated ability to manage five medications.
5. *Meal Planning*: Subjects follow recipes to simulate preparing a simple meal aiming to have three components ready at the same time [15]. Points are awarded for following instructions, and completing components simultaneously. Total score = 30 points.
6. . *Driving Assessment* using the DriveSafe DriveAware assessment [18]. An iPad application assessed awareness of driving environment and of personal performance. (Time ~ 10–15 min). Results are used to categorise participants as likely to pass a driving test, require further testing or likely to fail a driving test.

### Patient-reported outcomes

Functional Assessment of Cancer Therapy-Cognition (FACT-Cog) (version-3) [19] evaluating cognitive symptoms.

FACT-Fatigue [20, 21] evaluating fatigue and quality of life (QOL).

General Health Questionnaire-12 assessing anxiety and depression [22].

European Organisation for Research and Treatment of Cancer QLQ-C30 two items assessing cognitive symptoms [23].

Activities of Daily Living (ADL) [24] and Instrumental ADL (IADL) [25] to assess functional ability.

Perceived Stress Scale [26] to evaluate stress.

Colinet Morbidity Index [27] to assess comorbid illnesses.

### Blood results

Blood tests included complete blood count, creatinine, liver function tests, carcino-embryonic antigen, sex hormones, 10 cytokines (not yet analysed), C-Reactive Protein and markers of blood clotting. Apolipoprotein genotyping (Apo-E) was collected at baseline [13].

### Neuroimaging substudy

Participants with no contraindication to magnetic resonance imaging (MRI) were invited to undergo neuroimaging using a 3-Tesla GE MR750 Discovery scanner with the following sequences acquired:

***Structural MRI:*** Two high-resolution, 3D T1-weighted images (TR = 7.2 ms; TE = 2.8 ms; flip angle = 8°; section thickness = 0.9 mm; number of slices = 196; FOV = 220 mm; matrix = 256; total time = 7 min) for quantitation of total brain and subcortical volumes. Total scan time 15 min.

***Proton Magnetic Resonance Spectroscopy (***^***1***^***H-MRS)*** to explore whether cancer survivors had different brain metabolic profiles compared to non-cancer patients that may be markers of accelerated ageing. Single voxel ^1^H-MRS was obtained using Point RESolved Spectroscopy (PRESS) acquisition with two chemical shift-selective imaging pulses for water suppression (TE = 35 ms; TR = 2000 ms; 128 acquisitions). The following voxel locations were sampled separately: (i) left hippocampus (voxel size = 2 × 3x1.5) and (ii) anterior cingulate cortex (voxel size = 2 × 2x2). Total scan time 10 min.

### Neuropsychological test scoring

Raw neuropsychological test scores were converted to demographically uncorrected scaled scores and then into T-scores corrected for age, education and gender [5, 13]. Follow-up scores were corrected for practice effect using published norms [28]. A mean T-score was calculated for each cognitive domain and the primary endpoint was a composite score representing global performance across the test battery. T-scores were also transformed into deficit scores (0–5 ranging from no to severe impairment) that were averaged to provide the global deficit score (GDS), grading overall impairment [29]. Cognitive impairment was defined as (i) a GDS of > 0.5 [29] (the first secondary outcome) and (ii) the ICCTF definition of impairment (> 1.5 SD below the normative mean on > 2 tests or > 2 SD on one test) [30]. Functional domain deficit scores were averaged to derive a global functional deficit score (FDS). A clinically significant FDS was defined as a FDS ≥ 0.52 [15].

## Statistical analysis

Baseline characteristics were compared for the original study participants and those completing long-term follow-up, using χ2 and Wilcoxon rank sum tests, to assess patterns of loss and generalisability of results.

Cognitive and functional performance were compared between CRC survivors and controls for mean T-scores and impairment status at baseline and follow-up; sensitivity analyses were performed to explore robustness of the results. Linear mixed models were conducted with the demographically and practice effect-corrected mean T-score as the outcome, with multiple imputation and random effects to control for attrition [31]. Model 1 included groups (CRC survivors versus controls) and assessment period (time) as fixed effects, time and group as an interaction term and subject as a random effect. Model 2 included group, time and baseline mean T-scores as fixed effects, time and group as an interaction term and subject as a random effect. The primary model was repeated using cognitive impairment status (GDS > 0.5 and ICCTF definition) in place of the baseline mean T-score adjustment as those that participated in the longitudinal assessments had better cognitive performance at baseline than those who did not [5]. A secondary model to evaluate ageing and sex effects used demographically uncorrected mean scaled scores. Models were repeated for the four cognitive domain T-scores.

Patient-reported outcomes and blood test results were compared between groups at baseline and long-term follow-up. FACT-Cog total score and apo-lipoprotein genotyping status were built into the cognitive models. All *p* values and confidence intervals are 2-sided and, with caveats relating to multiplicity, reported as unadjusted values with statistical significance defined at the α = 0.05 level.

Neuroimaging structural analyses utilised the FMRIB Software Library (FSL) (Oxford UK). Tissue segmentation and volumetric measurements were obtained using semi-automated techniques based on the principles of the Active Shape and Appearance Models within a Bayesian framework as implemented by the “FAST” and “FIRST” algorithms in FSL. ^1^H-MRS was analysed with *LCModel*, using a basic set of 15 metabolites*.* Absolute metabolite concentrations for N-Acetyl Aspartate (NAA), MyoInositol (MI), Creatine (Cre), Choline (Chol), Glutamate (Glu) and Glutathione (GSH) were calculated using Water-Scaling as implemented in LCModel and corrected for grey-white matter content within the voxel in each subject. Poorly defined metabolite peaks were excluded from analysis.

Sample size was determined by the number of eligible CRC survivors who were available and gave consent, with intent to include the same number of controls. Assuming 25 participants/group and assessments taken at 4 time points, a two-sided, alpha = 0.05, linear mixed effect model would have > 90% power to detect a moderate effect size and > 60% power to detect a small effect size in the primary endpoint.

## Results

Overall 114 participants with CRC in the original study were from Australia. Twenty-five survivors participated in this follow-up study of whom 9 had received chemotherapy; reasons for non-participation were death (*n* = 27), CRC recurrence (*n* = 11), new primary cancer (*n* = 8), patient refusal or other reason (*n* = 43). Twenty-five controls from the original study consented to participate (Supplementary Fig. 1).

Table 1 provides characteristics of the two groups. Median age at follow-up was 72 years (range 56–83) for the CRC survivors and 68 (34–80) for the controls; 60% of the survivors and 44% of controls were men. The control group was added later in the original study, and mean time from baseline was 8.9 years (range 6.0–12.2) for the CRC survivors and 6.2 years (range 5.6–7.3) for the controls. Cancer survivors had a higher Colinet comorbidity score, even allowing for scoring 1-point due to prior malignancy. There was no difference between groups in performance status.

**Table 1:** Summary statistics of participants at long-term follow up (LTFU) assessment

Characteristic	Statistic	CRC Survivors	Controls	*P* value
N		25	25	
Age – years at LTFU	Median [range]	72 [56, 83]	68 [34, 80]	0.030
Age ≥ 65 years at LTFU	N (%)	19 (76%)	16 (64%)	0.35
Gender	N (%) Male	15 (60.0)	11 (44.0)	
Years of education	Mean (SD) [range]	13.6 (2.5) [10–19]	13.8 (3.1) [6–20]	0.52
Marital status N (%)	Married/common law Separated/divorced Single Widowed Unknown	14 (56.0) 6 (24.0) 2 (8.0) 2 (8.0) 1 (4.0)	17 (68.0) 1 (4.0) 5 (20.0) 2 (8.0) 0 (0)	0.19
Alcohol (glasses/day)	0–1 2–4 5 + Unknown	11 (44.0) 10 (40.0) 3 (12.0) 1 (4.0)	6 (24.0) 16 (64.0) 2 (8.0) 1 (4.0)	0.38
Smoking status N (%)	Never Former Current	14 (56.0) 11 (44.0) 0 (0.0)	15 (60.0) 8 (32.0) 2 (8.0)	0.36
Language N (%)	English not primary language	4 (16.0)	2 (8.0)	0.38
Chemotherapy N (%)	None 5-Flourouracil/Capecitabine FOLFOX	16 (64.0) 5 (20.0) 4 (16.0)		
Years to follow-up	Mean (SD) [range]	8.9 (1.7) [6.0–12.2]	6.2 (0.5) [5.6–7.3]	< 0.001
Colinet Morbidity Index	Mean (SD) Median [range]	6.1 (3.9) 6 [1–14]	3.4 (4.1) 1 [0–12]	0.004
ECOG Performance Status N (%)	ECOG 0 ECOG 1	20 (80%) 5 (20%)	21 (84%) 4 (16%)	0.71

CRC = colorectal cancer

ECOG = European Cooperative Oncology Group

FOLFOX = 5-Flourouracil and Oxaliplatin

SD = standard deviation

### Cognitive function

Those cancer survivors who participated in the extended follow-up study scored better on cognitive outcomes at their original baseline assessment than those who did not participate in the follow up study, with higher mean T-scores (51.7 vs 48.9 *p* = 0.006) and lower GDS scores (0.44 vs 0.74, *p* = 0.04), as well as lower rates of baseline cognitive impairment on GDS (18% vs 32%, *p* = 0.05) and ICCTF criteria (16% vs 43%, *p* = 0.02). Differences were seen mainly in processing speed and verbal learning and memory. They were also more likely to have English as their primary language (88% vs 75%) and to be older (median age 62 vs 58 years) than those who did not participate. There were no differences in cognitive symptoms assessed by the FACT-COG.

At baseline, there were no differences between cancer survivors and controls in demographically corrected mean T-score or in cognitive impairment rates (12% in both groups) (Table 2). There was also no difference in impairment rates by GDS or ICCTF criteria at long-term follow-up (Table 2). There was a non-significant trend for lower scores in all domains in CRC survivors but differences were small.

**Table 2:** Mean (SD) T-score of cognitive domains and individual neuropsychological tests and number (proportion) of participants with impairment ^a^ by study group and visit

Ability domain/test	Visit	CRC survivors	Controls	Difference (95% CI)
		Total		
		Mean score (SD)	Mean score (SD)	
MMSE	LTFU	28.6 (2.3) (19–30)	29.4 (0.87) (27–30)	− 0.8 (–1.9, 0.2)
WRAT-3 T-score (range)	LTFU	56.8 (6.1) (44–63)	58.3 (4.9) (41–63)	− 1.5 (–4.7, 1.6)
Global Deficit Score (SD)	Baseline	0.2 (0.6)	0.1 (0.6)	0.04 (–0.27, 0.35)
	LTFU	0.2 (0.6)	0.1 (0.4)	0.13 (–0.14, 0.38)
Mean T-score (SD)	Baseline	50.5 (7.4) (34–65) 53.0 (9.6) (33–70)	53.6 (7.4) (28–62) 56.1 (8.0) (34–67)	− 3.1 (–7.4, 1.1) − 3.1 (–8.3, 1.9)
Cognitive Impairment GDS criteria ICCTF criteria	Baseline Baseline	N (%) 3 (12%) 4 (16%) 3 (12%) 5 (20%)	N (%) 3 (12%) 2 (8%) 2 (8%) 2 (8%)	0 (–18, 18) 8 (–14, 30) 4 (–17, 25) 12 (–11, 35)
Clinical cognitive domains		Mean T-score (SD)	Mean T-score (SD)	
Attention and working memory domain	Baseline LTFU	53.2 (8.8) 56.0 (10.7	53.7 (6.2) 57.1 (7.1)	− 0.5 (− 4.8, 3.89) − 1.1 (− 6.3, 4.1)
Processing speed domain	Baseline LTFU	50.5 (7.8) 53.6 (9.9)	55.1 (9.3) 57.7 (9.8)	− 4.7 (− 9.5, 0.2) − 4.1 (− 9.7, 1.5)
Verbal learning and memory domain	Baseline LTFU	46.7 (9.9) 50.0 (10.7)	50.3 (10.7) 54.0 (8.1)	− 3.7 (− 9.5, 2.2) − 4.0 (− 9.4, 1.4)
Visual learning and memory domain	Baseline LTFU	50.1 (12.8) 52.3 (13.7)	53.5 (12.3) 55.5 (13.4)	− 3.4 (− 10.6, 3.7) − 3.2 (− 10.9, 4.5)
Functional deficit score mean (SD) (range)	LTFU	0.43 (1.01) (0–4.3)	0.30 (0.51) (0–1.7)	0.13 (− 0.10, 0.45)
Grooved pegboard mean (SD) Dominant hand Non-dominant hand	LTFU	135.0 (57.9) 140.1 (49.4)	113.9 (31.3) 117.8 (31.6)	21.0 (− 5.6, 47.7) 22.3 (− 1.8, 46.4)

^a^ Cognitive impairment defined by:

Global Deficit Score (GDS) > 0.5

International Cognition and Cancer Task Force (ICCTF) criteria

*LTFU* long-term follow-up, *SD* standard deviation

**Fig. 1:**
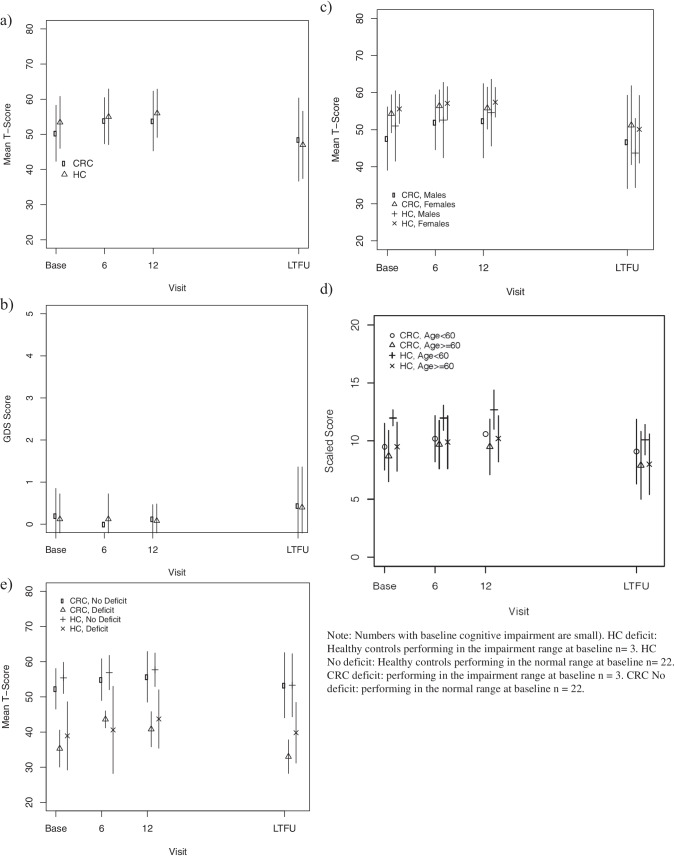
Longitudinal cognitive scores (mean ± standard deviation) by group (colorectal cancer survivors vs controls). **a** Mean T-score—demographically and practice effect corrected. **b **Global Deficit Score—demographically and practice effect corrected. **c** Mean T-score by sex—demographically and practice effect corrected. **d** Scaled score by age (> 60 years, ≤ 60 years). **e** Mean T-score based on whether or not baseline cognitive impairment was present (GDS > 0.5)—demographically and practice effect corrected

There were no differences over time in demographically and practice effect-corrected mean T-scores between cancer survivors and controls (see Table 3, Supplementary Table 2, and Fig. 1). Cognitive performance at baseline predicted cognitive function scores at follow-up. The four cognitive domains showed no consistent difference between groups (Supplementary Table 3). We found no evidence of accelerated ageing in survivors of CRC using demographically uncorrected mean scaled scores and no significant effect of sex (Table 4).

**Table 3:** Mixed effect model results comparing CRC survivors to controls over time adjusting for a) baseline performance and b) baseline Global Deficit Scores (GDS)

Outcome: demographically and practice effect corrected mean T-score			
a)			
Parameter	Estimate	SE	*P* value
Intercept	7.61	5.99	*NS*
CRC survivors Group effect	− 3.90	8.93	*NS*
Time effect			
6 months 12 months 24 months	1.42 6.05 6.00	6.66 6.66 6.74	*NS* *NS* *NS*
Group* Time			
6 months 12 months	1.40 − 1.31	9.89 9.48	*NS* *NS*
Baseline T-score	0.91	0.11	< 0.0001
Baseline T-score* Time			
6 months 12 months 24 months	− 0.05 − 0.11 − 0.08	0.13 0.13 0.13	*NS* *NS* *NS*
Baseline T-score*Group* Time			
6 months 12 months	0.00 0.05	0.19 0.18	*NS* *NS*
b)			
Outcome: demographically and practice effect corrected mean T-score			
Parameter	Estimate	SE	*P* value
Intercept	0.125	0.05	0.02
CRC survivors group effect	0.09	0.09	*NS*
Time effect			
6 months 12 months 24 months	− 0.125 − 0.125 0.20	0.09 0.09 0.09	*NS* *NS* 0.04
Group* Time			
6 months 12 months	− 0.05 − 0.03	0.13 0.13	*NS* *NS*
Baseline Global Deficit Score (GDS)	0.96	0.09	< 0.0001
Baseline GDS* Time			
6 months 12 months 24 months	0.04 − 0.29 − 0.27	0.15 0.15 0.14	*NS* 0.05 0.05
Baseline GDS*Group* Time			
6 months 12 months	− 1.27 − 0.002	0.33 0.20	0.0002 *NS*

*NS* not significant

Where group is included data for 24-assessment are not available as the control group was not assessed at 24-months

**Table 4:** Mixed effect model results on the demographically uncorrected mean scaled scores comparing CRC survivors to controls assessing an age and an age*time effect (accelerated ageing) and age*group*time effect (accelerated ageing by group)

Outcome: demographically uncorrected mean scaled scores			
Parameter	Estimate	SE	*P* value
Intercept	17.94	2.41	< 0.001
CRCs Group effect	2.87	3.93	*NS*
Time effect			
6 months 12 months	-1.59 -0.61	1.73 1.4	*NS* *NS*
Group* Time			
6 month	-2.16	1.74	*NS*
Age (at LTFU)	-0.11	0.035	0.004
Age*Group (CRCs)	-0.04	0.06	*NS*
Age* Time			
6 months 12 months	0.02 0.01	0.02 0.02	*NS* *NS*
Age*Group* Time			
6 months	0.03	0.02	*NS*

*CRCs* colorectal cancer survivors, *LTFU* long-term follow-up, *NS* not significant

24 month assessments were not completed by the control group. Some estimates of multiple group interactions are blank as they are linear combinations of available estimates

There was no significant difference between groups in function: the mean global FDS scores were 0.4 (range 0–4.3) in CRC survivors versus 0.3 (0–1.7) in controls (*p* = 0.86). Based on our criteria, 4/24 (16.7%) of CRC survivors were impaired on functional tasks compared to 4/25 (16%) of controls (Table 2 and Supplementary Table 4).

There was no significant difference in cognitive symptoms in CRC survivors compared to controls (Supplemental Table 5). No association was seen between the total FACT-COG score or FACT-COG perceived cognitive impairment subscale (evaluating cognitive symptoms) and mean total T-score adjusted for group and time (evaluating cognitive function). No difference was found between the groups in global QOL, symptoms of anxiety/depression, stress, ADL or iADL. CRC survivors had a non-significant trend to experiencing more fatigue than controls at original evaluation and long-term follow-up.

**Table 5:** Neuroimaging results (mean and standard deviation) for brain metabolites and brain volumes

Test	Statistic	Controls	Colorectal Cancer Survivors	Wilcoxon rank sum test *P* value
N		8	10	
Brain metabolites				
Gamma-aminobutyric acid	Mean (sd)	1.87 (0.18)	1.86 (0.22)	*NS*
N-Acetyl Aspartate	Mean (sd)	8.18 (0.92)	8.17 (1.59)	*NS*
Glutamate	Mean (sd)	8.54 (0.96)	8.21 (1.71)	*NS*
Creatine	Mean (sd)	7.05 (0.69)	7.25 (0.77)	*NS*
Choline	Mean (sd)	2.08 (0.33)	2.23 (0.26)	*NS*
MyoInositol	Mean (sd)	4.38 (0.62)	5.32 (0.47)	0.012
Brain Volumes (mm^3^)				
L Amygdala	Mean (sd)	1354 (216)	1169 (265)	*NS*
R Amygdala	Mean (sd)	1412 (196)	1064 (147)	0.01
L Caudate	Mean (sd)	3215 (412)	3043 (282)	*NS*
R Caudate	Mean (sd)	3678 (487)	3102 (309)	0.015
L Hippocampus	Mean (sd)	3486 (279)	2935 (305)	0.005
R Hippocampus	Mean (sd)	3305 (284)	3138 (333)	*NS*
L Pallidus	Mean (sd)	1650 (311)	1588 (278)	*NS*
R Pallidus	Mean (sd)	1535 (151)	1392 (152)	*NS*
L Thallamus	Mean (sd)	7011 (830)	6500 (780)	*NS*
R Thallamus	Mean (sd)	6880 (719)	6236 (664)	*NS*

*L* left, *R* right, *NS* not significant

### Neuroimaging

Neuroimaging was completed in 18 participants (10 CRCs and 8 controls). The only metabolite concentrations with significant differences in levels between the groups was MI which was higher in CRC survivors than controls (*p* = 0.012). There was significantly less volume in the right amygdala and caudate and left hippocampal regions in the CRC survivors compared to controls (Table 5).

### Blood tests

There were no significant differences in results of blood tests between cancer survivors and controls. No participants were homozygous for ApoE4, but 4 cancer survivors and 9 controls had one ApoE4 allele. Presence of an E4 allele had no significant effect on mean neuropsychological T-score but was associated with significantly higher GDS (*p* = 0.01), and at the cognitive domain level, with poorer attention/executive function (*p* = 0.001), and trends towards lower verbal and visual memory performance.

## Discussion

We found no significant differences in cognitive, functional or patient reported outcomes between CRC survivors and controls at 6–12 years after diagnosis and no evidence of accelerated ageing. Baseline cognitive performance was the only significant predictor of cognitive performance at follow-up; this result is not surprising and has been observed in other clinical populations [32, 33].

We are unaware of other cognitive studies with long-term follow up of CRC survivors. A recent study of women diagnosed with breast cancer aged over 55 years designed to assess participants 5–15 years later found impairments only in learning and memory domains compared to non-cancer controls [34]. No differences in cognitive domain scores were found in survivors who received chemotherapy compared to those who did not. Cognitive symptoms were also similar between survivors and controls, although cancer survivors had significantly higher frailty scores, which were associated with poorer cognitive performance.

Another study that assessed women ~ 21 years after receiving adjuvant chemotherapy for breast cancer also performed neuroimaging [7]. Breast cancer survivors had smaller total brain volume and grey matter volume than the control participants [35]. No significant differences were seen in white matter integrity but there was an inverse association between time from chemotherapy and lower global and focal white matter integrity, suggesting deterioration in white matter integrity over time [36]. We also found differences in brain volume in our CRC survivors, with less volume in the right amygdala and caudate and left hippocampal regions, compared to controls. Reduced grey matter in people with cancer has been reported in several studies, including prior to diagnosis [37]. However, a large population-based study with 353 participants diagnosed subsequently with cancer found no difference in brain abnormalities prior to diagnosis compared to people who remained cancer free [38]. A systematic review of 14 longitudinal studies (11 in breast cancer) in 560 adults who had received chemotherapy found reduced grey matter density in frontal, parietal and temporal regions, changes in brain function, reduced white matter integrity and changes in brain structural connectivity [37]. Follow-up neuroimaging generally was conducted within a few months of completing chemotherapy and the average age of participants was 48 years. Studies with longer-term follow up are required to determine changes over time.

Studies in non-cancer patients with mild cognitive impairment consistently report significant decreases in *N*-acetyl aspartate levels [39], but increased myo-inositol [40]. Oxidative stress has been proposed to contribute to the development of cognitive impairment, with significant changes in glutathione reported in mild cognitive impairment [41]. Our neuroimaging study was underpowered but did find significantly higher myo-inositol levels in long-term CRC survivors than controls.

The *Thinking and Living with Cancer* study followed women diagnosed with early-stage breast cancer (*n* = 344) and matched controls (*n* = 347) aged 60 years or older for 2 years and reported that survivors who received chemotherapy had more impairment in the domains of attention, processing speed and executive function [42]. This effect was predominantly seen in women with an apoE4 allele. Epsilon 2 genotype was found to be protective [43].

Analysis of ApoE4 allele in our sample is underpowered but suggests a trend for those with an ApoE4 allele to have lower cognitive scores. Other studies show mixed results but most are limited by sample size. Ahles et al. found ApoE4 conferred increased cognitive impairment in long-term survivors of breast cancer and lymphoma [44]. Amidi et al. reported poorer cognitive functioning in testicular cancer patients with the ApoE4 allele but no correlation with ApoE4 genotype and changes in brain structure [45]. McDonald et al. found no association between ApoE4 genotype and a decrease in frontal grey matter density or with executive function in breast cancer survivors 1 month after completing chemotherapy [46].

## Strengths and limitations

The main strength of our study is analysis of longitudinal data from soon after diagnosis to 6–12 years later in men and women with CRC and in matched non-cancer controls. The relatively small sample available for long-term follow-up assessment limited the power of the study to detect differences in cognitive performance and did not allow comparison of those who did and did not receive chemotherapy. Those who participated had better cognitive performance at baseline compared to the original cohort and are not therefore a random sample. To address this, we used baseline performance and cognitive impairment as predictors in the linear mixed effect regression models but recognize that this is an imperfect solution. The global deficit score is designed to focus on below normal performance, giving less weight to performance within and above normal limits. This means participants could have a marked decline from an above average GDS but if they remain within the normal range would not be classified as impaired. Inclusion of neuroimaging adds to our study but interpretation is limited by lack of baseline assessment and relatively small numbers. All of our results should be regarded as exploratory and hypothesis-generating.

## Supplementary Information

Below is the link to the electronic supplementary material.

Supplementary file1 (DOCX 64 KB)

## Data Availability

Data will be shared on reasonable request to the corresponding author.
