# Supplementary material for: Lack of cognitive impairment in long-term survivors of colorectal cancer
Source: Support Care Cancer. 2022 Apr 14;30(7):6123–33. doi: 10.1007/s00520-022-07008-3 (PMC9135780; doi:10.1007/s00520-022-07008-3)
Supplement: Supplementary file 1 — Supplementary file1 (DOCX 64 KB) [file 520_2022_7008_MOESM1_ESM.docx]

**Supplementary Table 1. Neuropsychological Assessment**

| Domains Assessed: | Tests |
| --- | --- |
| Screening test | Mini Mental State Examination (MMSE):*  Subjects scoring <18/30 to perform as much of the study assessments as they are able to and classified as having dementia. |
| Intellectual ability | Wide Range Achievement Test (WRAT) 3 Reading test:*  -assessing premorbid intellectual ability. |
| Attention/ Working Memory | Wechsler Adult Intelligence Scale (WAIS) III Letter-Number  Sequencing  Wechsler Memory Scale (WMS) III Digit Span  WMS-III Spatial Span  Stroop Colour and Word* |
| Processing speed | Symbol Digit Modalities Test  Trail Making Test Part A Trail Making Test Part B |
| Verbal Learning and Memory | Hopkins Verbal Learning Test-Revised –(HVLT-R) |
| Visual Learning and Memory | Brief Visuospatial Memory Test-Revised – (BVMT-R) |
| Verbal Fluency | Controlled Oral Word Association (COWA)* |

*Tests not included in mixed models as were not included in the original study.

**Supplementary Table 2. Mixed effect model results on the demographically and practice effect corrected T-scores comparing CRC survivors to controls over time adjusting for baseline cognitive impairment defined by a) global deficit score (GDS) >0.5 and b) International Cognition and cancer Task Force (ICCTF) criteria**

| Outcome: demographically and practice effect corrected mean T-score | | | |
| --- | --- | --- | --- |
| Parameter | Estimate | SE | P-value |
| Intercept | 42.84 | 3.07 | <0.0001 |
| Group | -5.50 | 4.39 | *NS* |
| Time:  6-month  12-month  24-month | 0.46  2.54  4.65 | 2.33  2.33  2.33 | *NS*  *NS*  *NS* |
| Group*Time:  6-month  12-month | 5.91 3.07 | 3.55  3.30 | 0.09  *NS* |
| GDS impairment | 14.31 | 3.28 | <0.0001 |
| Group*GDS impairment | 2.69 | 4.68 | *NS* |
| GDS impairment*Time:  6-month  12-month  24-month | -2.06  -2.88  -3.10 | 2.48  2.48  2.49 | *NS*  *NS*  *NS* |
| GDS impairment*Group*Time:  6-month  12-month | -4.80  -1.63 | 3.76  3.52 | *NS*  *NS* |

| Outcome: demographically and practice effect corrected mean T-score | | | |
| --- | --- | --- | --- |
| Parameter | Estimate | SE | P-value |
| Intercept | 49.78 | 4.46 | <0.0001 |
| Group | -8.26 | 5.53 | *NS* |
| Time:  6-month  12-month  24-month | -5.80  -2.73  0.07 | 2.89  2.89  2.36 | 0.05  *NS*  *NS* |
| Group* Time:  6-month  12-month | 7.67  3.32 | 3.97  3.74 | 0.06  *NS* |
| ICCTF Impairment | 6.14 | 4.65 | *NS* |
| Group* Impairment | 6.11 | 5.81 | *NS* |
| Time* Impairment  6-month  12-month  24-month | 4.83  2.98  2.12 | 3.02  3.02  2.52 | *NS*  *NS*  *NS* |
| Group* Time* Impairment  6-month  12-month | -6.62  -1.76 | 4.16  3.93 | *NS*  *NS* |

NS = not significant

ICCTF criteria for classification of impairment: < 1.5 SD below the normative mean on >2 tests or >2 SD on one test. Ingraham and Aiken equation used to consider the influence of multiple tests.^30^

Note: where group is included data for 24-assessments are not available as the control group were not assessed at 24-months

**Supplementary Table 3. Mixed effect model results on the demographically and practice effect corrected mean T-scores for cognitive domains comparing CRC survivors to controls over time adjusting for baseline mean cognitive domain T-scores**

1. Attention/executive function

| Outcome: demographically and practice effect corrected mean T-scores | | | |
| --- | --- | --- | --- |
| Parameter | Estimate | SE | P-value |
| Intercept | 10.5572 | 8.6368 | *NS* |
| CRC survivors Group effect | -9.1635 | 9.9153 | *NS* |
| Time effect:  6-month  12-month  24-month | 5.0770  1.4189  -1.1269 | 7.5879  7.5879  4.6783 | *NS*  *NS*  *NS* |
| Group* Time:  6-month  12-month | -0.9112  3.1066 | 9.1502  8.9141 | *NS*  *NS* |
| Baseline attention/executive T-score | 0.8655 | 0.1566 | <0.0001 |
| Baseline T-score* Time:  6-month  12-month  24-month | -0.1017  -0.01053  0.02670 | 0.1375  0.1375  0.08516 | *NS*  *NS*  *NS* |
| Baseline T-score*Group* Time:  6-month  12-month | -0.01440  -0.08360 | 0.1654  0.1617 | *NS*  *NS* |

1. Processing speed

| Outcome: demographically and practice effect corrected mean T-scores | | | |
| --- | --- | --- | --- |
| Parameter | Estimate | SE | P-value |
| Intercept | 5.9678 | 4.9503 | *NS* |
| CRCs Group effect | -6.7622 | 7.4851 | *NS* |
| Time effect:  6-month  12-month  24-month | 1.2287  3.6043  12.268 | 5.0711  5.0711  5.522 | *NS*  *NS*  0.03 |
| Group* Time:  6-month  12-month | 11.3838  5.0577 | 7.7893  7.4976 | *NS*  *NS* |
| Baseline processing speed T-score | 0.9391 | 0.08862 | <0.0001 |
| Baseline T-score* Time:  6-month  12-month  24-month | -0.02204  -0.03445  -0.1786 | 0.09078  0.09078  0.1082 | *NS*  *NS*  *NS* |
| Baseline T-score*Group* Time:  6-month  12-month | -0.2392  -0.1170 | 0.1464  0.1413 | *NS*  *NS* |

1. Verbal Memory Domain

| Outcome: demographically and practice effect corrected mean T-scores | | | |
| --- | --- | --- | --- |
| Parameter | Estimate | SE | P-value |
| Intercept | 19.44 | 4.18 | <0.0001 |
| CRCs Group effect | 9.3865 | 7.6122 | *NS* |
| Time effect:  6-month  12-month  24-month | -5.7361  -8.4461  -4.4495 | 7.2379  7.2379  7.4051 | *NS*  *NS*  *NS* |
| Group* Time:  6-month  12-month | -10.5751  -8.1533 | 10.5677  10.3548 | *NS*  *NS* |
| Baseline Verbal memory T-score | 0.6861 | 0.08131 | <.0001 |
| Baseline T-score* Time:  6-month  12-month  24-month | 0.07186  0.1742  0.1453 | 0.1408  0.1408  0.1542 | *NS*  *NS*  *NS* |
| Baseline T-score*Group* Time:  6-month  12-month | 0.3106  0.2189 | 0.2124  0.2088 | *NS*  *NS* |

1. Visual Memory

| \| Outcome: demographically and practice effect corrected mean T-scores \| \| --- \| | | | |
| --- | --- | --- | --- | --- |
| Parameter | Estimate | SE | P-value |
| Intercept | 14.50 | 6.82 | 0.04 |
| CRCs Group effect | 2.93 | 8.89 | *NS* |
| Time effect:  6-month  12-month  24-month | -1.43  11.30  4.25 | 6.95  6.95  6.29 | *NS*  *NS*  *NS* |
| Group* Time:  6-month  12-month | 12.69  3.12 | 10.41  9.37 | *NS*  *NS* |
| Baseline Visual Memory T-score | 0.77 | 0.12 | <.0001 |
| Baseline T-score* Time:  6-month  12-month  24-month | -0.02  -0.28  -0.10 | 0.13  0.13  0.12 | *NS*  0.03  *NS* |
| Baseline T-score*Group* Time:  6-month  12-month | -0.19  -0.02 | 0.19  0.18 | *NS*  *NS* |

Note: where group is included data for 24-assessment are not available as the control group did not do 24-month assessments

NS = not significant

**Supplementary Table 4. Mean, standard deviation and range on functional deficit scores (FDS), number impaired on FDS and individual functional task scores**

Mean (SD)/range

| Domains | | CRC survivors  (N=17-24)* | | Controls  (N=18-25)* | |  |
| --- | --- | --- | --- | --- | --- | --- |
|  | |  | |  | |  |
| FDS | | 0.43 (1.01)  (0-4.3) | | 0.30 (0.51)  (0-1.7) | |  |
| Impaired on FDS n (%) | | 4/24 (17%) | | 4/25 (16%) | |  |
| Finances | | 40.1 (2.3)  (32-42) | | 38.4 (4.2)  (23-42) | |  |
| Shopping | | 15.8 (3.0)  (8-20) | | 16.2 (2.4)  (9-20) | |  |
| Meal planning and preparation | | 25.4 (3.6)  (15-30) | | 26.2 (3.5)  (20-30) | |  |
| Medication management | | 14.8 (3.5)  (4-17) | | 15.8 (1.9)  (10-17) | |  |
| Driving: N (%)    Likely to pass on road test  Require further testing  Likely to fail on road test | | 24^  21 (84%)  2 (8%)  2 (8%) | | 25  23 (92%)  1 (4%)  1 (4%) | |  |
|  |  |  |  | |  |  |
|  |  |  |  | |  |  |

^ One colorectal cancer (CRC) survivor had never had a driver’s licence and was excluded from this task.

**Supplementary Table. 5 Mean (standard deviation) of patient reported outcomes of baseline assessment and long term follow up comparing colorectal cancer (CRC) survivors to controls**

| Questionnaire | Visit | CRC survivors  Mean (SD) [range] | Controls  Mean (SD) [range] | Difference  (95% confidence interval) |
| --- | --- | --- | --- | --- |
| FACT-Cog:  Total    PCI    PCA  CogQOL  CogOthers | Baseline  LTFU  Baseline  LTFU  Baseline  LTFU  Baseline  LTFU  Baseline  LTFU | 108.4 (16.8)  112.7 (14.0)  60.8 (9.5)  62.1 (8.5)  18.8 (5.0)  15.2 (1.1)  14.3 (3.1)  20.3 (7.0)  15.4 (2.1)  14.7 (2.2) | 112.6 (13.0)  116.1 (12.5)  59.8 (8.1)  63.6 (7.0)  22.6 (5.0)  15.6 (0.6)  15.7 (0.7)  21.8 (6.1)  14.6 (1.6)  15.4 (1.3) | 4.3 (-4.3 - 12.8)  3.4 (-4.2 – 10.9)  -1.1 (-6.1 – 4.0)  1.5 (-2.9 – 6.0)  3.7 (-0.2 – 7.6)  -0.4 (-0.2 – 7.6)  1.4 (0.1 – 2.7)  1.5 (0.1 – 2.7)  0.9 (-0.2 – 1.9)  0.7 (-03 – 1.7) |
| EORTC-CF: (scaled 0-100) | LTFU | 13.3 (10.8)  [0 - 33.3] | 13.3 (12.7)  [0 - 33.3] | 0 (-6.7 - 6.7) |
| ADL (SD)  [range] | LTFU | 20.7 (0.5)  [19 – 21] | 21 (0.2)  [20 – 21] | -0.24 (-0.48 - -0.004) |
| iADL (SD)  [range] | LTFU | 8 (0)  [8 – 8] | 8 (0)  [8 - 8] | 0 (-) |
| FACT-F subscale | Baseline  LTFU | 41.8 (8.3)  40.4 (3.4) | 45 0 (6.8)  42.3 (3.8) | 3.2 (-1.1 – 7.6)  1.9 (-0.2 – 3.9) |
| FACT-G | Baseline  LTFU | 86.6 (12.7)  91.6 (12.4) | 93.6 (11.0)  90.3 (7.7) | 7.0 (0.1 -14.0)  -1.3 (-7.2 – 4.6) |
| GHQ | Baseline  LTFU | 9.7(3.4)  8.4 (2.3) | 8.8 (2.5)  9.0 (3.4) | -0.9 (-2.6 – 0.8)  0.6 (-1.1 – 2.2) |
| PSS | LTFU | 28 (8.7) | 28.4 (8.8) | -0.44 (-5.4, 4.5) |

FACT = Functional Assessment of Cancer Treatment; Cog = cognition v3; F = fatigue subscale; G = general. FACT scores – higher score represents less symptoms (more ability).

PCI = Perceived cognitive impairments; PCA = perceived cognitive abilities; CogQOL = impact of cognition on QOL; CogOthers = comments from others regarding cognition.

EORTC-CF = European Organisation for Research and Treatment of Cancer- Cognitive Functioning. Scaled 0-100 (lower score represents less symptoms).

GHQ = General Health Questionnaire 12 (higher score represents more symptoms).

PSS= Perceived Stress Scale (higher score represents higher levels of stress).

SD = standard deviation

**Supplementary Figure 1 Consort diagram**

Mailed invitation letter n = 143

Localised CRC:

Chemotherapy n = 32

No chemotherapy n = 42

Controls n =69

Control n = 25

CRC survivors:

Chemotherapy n = 9

No chemotherapy n = 16

Consented n = 51

Non-participation n= 92

Reason:

Not interested in study n = 5

Too busy n = 11

Carer duties n = 1

Poor health n = 6

Moved out of Sydney n = 4

Recurrence/progression of CRC n = 4

New primary cancer n = 3

Unable to contact n = 17

Excess control participants n = 41

Assessed for eligibility for long-term follow up study (n = 186)

(excludes metastatic group)

Not eligible or not available n = 43

Reason:

Deceased n = 26

Recurrence n = 9

New primary cancer n = 5

Moved out of Sydney n = 3

Deceased between consent and assessment n = 1 CRC survivor

Australian participants from original study (n=199)

Localised CRCs n =144

(Chemotherapy n=56;

No chemotherapy n=58)

Metastatic CRC n =13

Controls n =72
